# The effects of *Berberis vulgaris* consumption on plasma levels of IGF-1, IGFBPs, PPAR-γ and the expression of angiogenic genes in women with benign breast disease: a randomized controlled clinical trial

**DOI:** 10.1186/s12906-019-2715-1

**Published:** 2019-11-21

**Authors:** Saeed Pirouzpanah, Sanaz Asemani, Ali Shayanfar, Behzad Baradaran, Vahid Montazeri

**Affiliations:** 1 Nutrition Research Center, Tabriz University of Medical Sciences, Tabriz, 5166614711 Iran; 2 Drug Applied Research Center, Tabriz University of Medical Sciences, Tabriz, Iran; 3 Molecular Medicine Research Center, Biomedicine Institute, Tabriz University of Medical Sciences, Tabriz, Iran; 4 Pharmaceutical Analysis Research Center, Tabriz University of Medical Sciences, Tabriz, Iran; 5 Faculty of Pharmacy, Tabriz University of Medical Sciences, Tabriz, Iran; 6 Immunology Research Center, Tabriz University of Medical Sciences, Tabriz, Iran; 7 Department of Thoracic Surgery, Faculty of Medicine, Tabriz University of Medical Sciences/ and also Surgery Ward, Nour-Nejat Hospital, Tabriz, Iran

**Keywords:** Benign breast disease, *Berberis vulgaris*, Insulin-like growth factor, *PPAR*, *VEGF*, *HIF*

## Abstract

**Background:**

The present study was designed to investigate the effects of *Berberis vulgaris* (*BV*) juice consumption on plasma levels of insulin-like growth factor (IGF-1), IGF-binding proteins (IGFBPs), and the expression of *PPAR-γ*, *VEGF* and *HIF* in women with benign breast disease.

**Methods:**

This parallel design randomized, double-blind controlled clinical trial was conducted on 85 eligible patients diagnosed with benign breast disease. They were assigned randomly into either *BV* juice group (*n* = 44, BV juice: 480 ml/day) or placebo group (*n* = 41, *BV* placebo juice: 480 ml/day) for 8 weeks intervention. Participants, caregivers and those who assessed laboratory analyses were blinded to the assignments. Plasma levels of biomarkers were measured at baseline and after 8 weeks by ELISA. Quantitative real-time PCR was used to measure the fold change in the expression of each interested gene.

**Results:**

The compliance of participants was 95.2% and 40 available subjects analyzed in each group at last. Relative treatment (RT) effects for BV juice caused 16% fall in IGF-1 concentration and 37% reduction in the ratio of IGF-1/1GFBP1. Absolute treatment effect expressed 111 ng/ml increased mean differences of IGFBP-3 between *BV* group and placebo. Plasma level of *PPAR-γ* increased in both groups but it was not significant. Fold changes in the expressions of *PPAR-γ*, *VEGF* and *HIF* showed down-regulation in the intervention group compared to placebos (*P* < 0.05).

**Conclusions:**

The *BV* juice intervention over 8 weeks was accompanied by acceptable efficacy and decreased plasma IGF-1, and IGF-1/IGFBP-1 ratio partly could be assigned to enhanced IGFBP-1 level in women with BBD. The intervention caused reductions in the expression levels of *PPAR, VEGF,* and *HIF* which are remarkable genomic changes to potentially prevent breast tumorigenesis.

**Trial registration:**

IRCT2012110511335N2. Registered 10 July 2013 (retrospectively registered).

## Introduction

Benign breast disease (BBD) is heterogeneous groups of pathologic lesions [1], includes three main histologic entities: 1) non-proliferative lesion, 2) proliferative lesion without atypia, and 3) atypical hyperplasia [2]. Generally, the proliferative BBD is a pathologic lesion highly susceptible to transform into malignancy [3], might elevate 50 % risk of BC incidence, whereas those with cellular atypia are at almost 4-fold increased risk for BC incidence at later in life [3]. Therefore, BBD is recently implicated as a major health public concern due to the high prevalence rate of subsequent breast cancer as the consequence of previously pathologic diagnosed BBD [1].

Insulin-like growth factor-1 (IGF-1) is a hormone with anti-apoptotic and mitogenic potentials correlated to the tumor growth and progression toward malignancy [4]. Unlike insulin, IGF-1 is widely transcribed by many cells such as transformed tumoral cells, expressing the diverse sources of the IGF-1 expression, therefore acts as an autocrine growth factor [3]. IGF-binding protein-3 (IGFBP-3) and IGFBP-1 bind in a ternary complex with circulating IGF-1, both lead to lower free form in circulation and subsequent less actively available ligand for mitogen-activated protein kinase (MAPK) (Ras/Raf/MEK/ERK) signaling pathway to promote IGF-dependent tumorigenesis [5]. Experimental studies have shown that IGFBP-3 can play apoptotic and anti-proliferative roles for normal breast cells in a dose-dependent manner [6]. In detail, low concentration of IGFBP-3 in normal breast cells has been reported to antagonize IGF-1 action and might, therefore, lead to inhibition on potential tumorigenicity of IGF-1, whereas higher doses of IGFBP-3 may act different and promote cell growth [3]. High serum level of IGFBP-3 may stabilize IGF-1 and subsequently may hold IGF-1 pool reserved to potentiate tumorigenesis [7]. However, much less information available to show the IGFBP-response rate in association with dietary modalities.

Similar to IGFBP-3, IGFBP-1 is another carrier of IGF-1 that controls the homeostasis of free IGF-1 [8]. Primarily, there is a lack of explicit findings to show that IGFBP-1 evolves tumorigenesis, whereas it is widely taken into account in the regulatory mechanism underlying tumor growth inhibition [6]. Thereby, considering this integrative network as the molar ratio of IGF-1/IGFBP-1 could help clinical studies to interpret the IGF-dependent tumorigenic features [9]. The growth hormone is a main regulatory factor affects the transcription of IGFBP-3 [8]. By contrast, transcription of IGFBP-1 is regulated significantly by the metabolic status, and subsequently, there is a prominent balancing act between energy intake and IGFBP-1 variations [10]. Overall, far little information is available about the effects of dietary factors on IGFBP levels and the biomarkers corresponding tumorigenesis of benign neoplasm.

Peroxisome proliferator-activated receptor-γ (*PPAR-γ*) modulates the expression of many genes actively involved in regulating the cell cycle [11]. Specific ligands bind PPAR-γ protein can exert inhibition on the proliferation of human breast carcinoma cells [11]. In contrast, the persistent over-regulation of the *PPAR-γ* gene can also increase the likelihood of breast carcinogenesis [12]. The marked increases in tumoral *PPAR-γ* expression are evident from internal control as adjacent normal breast tissue [12]. Together, observational results regarding *PPAR-γ* transcription in association with breast cancer development are inconsistent, and likewise, the varied effects showed for ligands bind to *PPAR-γ* on arresting the cell cycle of tumors which are remained as the subject of intense researches. To date, no available data showed the PPAR-related effects of interventions in BBD.

Vascular endothelial growth factor (*VEGF)* is an iconic angiogenic parameter in tumor growth [13] acts as a transcriptional factor for the downstream expression of angiopoietins [13]. Pathologic angiogenesis occurs when *VEGF* over-expressed and angiopoietin-2 increased in favor of tumoral neovascularization [14]. Several studies showed that the expression level of *VEGF* is over-regulated in the benign and cancerous breast tissues [15], and potentially can facilitate the pathologic transformation by supporting tumor circulation [16]. Hypoxia-inducible factor *(HIF)* involved in upstream transcription of main angiogenic genes such as VEGF and angiopoietins [17]. *HIF-1a* is overexpressed once in tumorigenesis [17]. It is shown that breast cancer patients with elevated levels of *HIF* are at an increased risk of tumor growth and metastasis [18]. Although, the role of *PPAR-γ*, *VEGF,* and *HIF* have been shown in several cancers [18, 19], little is known about their genomic pattern in BBD.

*Berberis vulgaris* (*BV*) belongs to *Berberidaceae* (plant family), is a widely used herbal plant as alternative medicine [20]. Dry *BV* fruit is consumed often and involved in Iranian dietary habits as a well-known local food seasoning. Barbery is also consumed as fresh or brewed juice in herbal drinks [21]. Berberine, an isoquinoline plant alkaloid, is the main component of *BV* isolated from the fruit and root extract of this plant [20]. There is a growing body of evidence to support the concept that berberine can inhibit proliferation and thereby provides anti-tumoral effects against several cancer types, such as breast carcinoma [22, 23]. Although berberine has been revealed to exert apoptosis for glioma [23], and leukemia cells [24], it does not have toxicity against healthy normal cells in vitro, and therefore related efficacy could be assigned to evaluate the effects of *BV* juice drink in malignant patients [24]. In addition, berberine is a ligand for PPAR and thereby it may have modulating effects on the anti-tumorigenic function of berberine [25]. Numerous experimental studies show the effects of berberine on alterations of *VEGF* and *HIF* in different cell lines [26, 27], whereas there is a lack of a clinical trial to address the effect of *BV* intervention on the expression levels of genes known actively involved in the initiation of tumorigenesis.

The intervening effects of dietary factors on metabolic changes regarding IGF-1 levels as a growth-promoting factor and angiogenic regulating factors have not been noticed thus far. Therefore, the present double-blind randomized controlled clinical trial was conducted on women recently diagnosed with BBD to study the effects of daily *BV* juice consumption on plasma levels of IGF-1, IGFBP-3, IGFBP-1 and IGF-1 bioavailability indices in circulation and fold changes in the expressions of *PPAR-γ*, *VEGF* and *HIF*.

## Materials and methods

### Study design

**Fig. 1:**
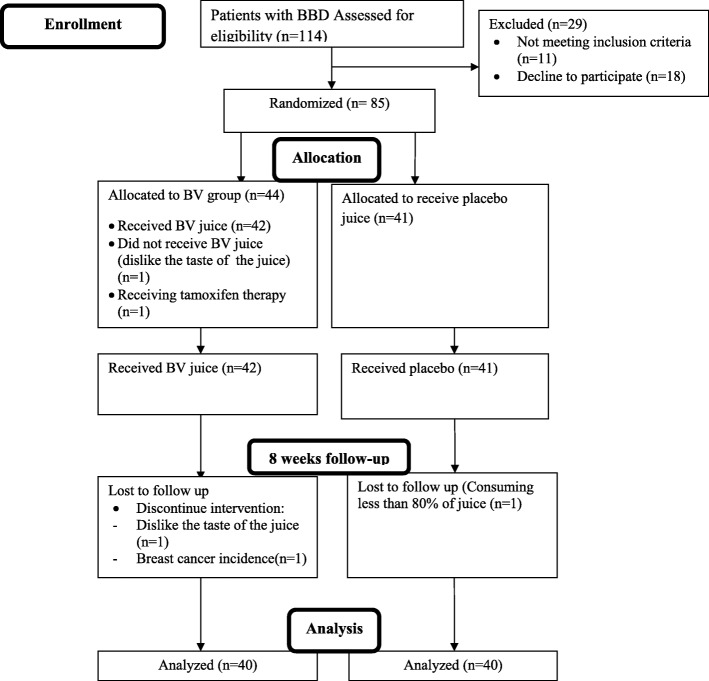
CONSORT flow diagram of the progress through different time compartments of a parallel randomized trial of two *BV* received and *BV* placebo received groups during 8 weeks of intervention. BBD, breast benign tumor disorder; *BV, Berberis vulgaris*

This randomized, double-blind, placebo-controlled clinical trial was conducted on 85 pre-menopausal women afflicted with BBD diagnosed by ultrasonography imaging aged between 19 and 52 years old (deviation to the considered age range in the protocol). They enrolled from May 2013 to October 2014 (retrospectively registered) who referred to the Nour-Nejat private hospital (Tabriz, Eastern Azerbaijan, Iran). The inclusion criteria were as follow: being premenopausal with fibrocystic changes or fibroadenoma, completed written informed consent, the interval of at least 2 years after the onset of the disease and no history of malignancy or benign neoplasm in any other anatomic sites. In addition, the subjects were excluded because of different reasons including: daily total caloric intake > 3500 kcal, history of active smokers, being pregnant or lactating mother when enrolled, gastrointestinal inflammatory disorders (peptic ulcer and gastritis), acute and chronic illnesses (including hyperthyroidism and other hormone-related disorder such as diabetes, polycystic ovary syndrome and hypoglycemia), clinical signs of bleeding, reported trauma (by patients or general practitioner), intolerance to *BV* juice, hormone replacement therapy (HRT, > a month), consuming medicines for insulin-resistant and anticoagulants drugs (such as aspirin). All the above-mentioned data were collected by a skillful questionnaire in a face-to-face interview. Pedigree analysis for each participant as a proband was questioned to obtain information regarding the positive family history of malignancies and benign neoplasms. The suitable sample size (*n* = 60) was estimated on the basis of prior data resulted in the study of Gu et al. [28], considered 5% level of significance and 20% false-negative error. By taking into account the possibility of less power of analysis for stratified random sequence generation (1:4 based on pathologic subtype; change to the method after trial commencement that had been 1:2), the number of enrolled participants was considered as high as 30% additional to the study protocol (Fig. 1). Finally, 85 women whose BBD recently diagnosed and fulfilled the selection criteria were randomly assigned into two groups via stratified random allocation. Computer-generated randomization software was used for allocation in the concept of parallel design. The study protocol was undertaken according to the revised guidelines released in the Declaration of Helsinki [29]. The trial protocol was submitted in Iranian Registry of Clinical Trials (IRCT) and received IRCT approval numbered in IRCT2012110511335N2. The IRCT is reflected in the World Health Organization (WHO) registry network set-up (www.irct.ir).

Finally, all eligible subjects were randomly allocated to receive an 8-weeks treatment of a daily 480 ml of either *BV* juice or placebo *BV* juice in a parallel design with superiority concept. The daily magnitude of juice consumption was a glass (serving size, 240 ml) in each meal at both lunch and dinner. Opaque white plastic bags contained tetra packs of juices were used to hand in for the duration of 2 weeks consumptions to achieve appropriate allocation concealment. For 7 days run-in period, participants were asked to consider consumption limits for anthocyanidin contained foods (such as sour cherry, cherry, blackberry, raspberry), other *Berberidaceae* (two serving unit a day), also alkaloid-contained spices (such as pepper and Sumac to a half teaspoon a day) and flavonoid-rich dietary products (a medium-sized apple, two cups of tea, one bowl of dried fruits a day). Each participant was also asked to follow these instructions in the run-in period by describing a study in easy ways to help them follow easily run-ins during the intervention. Although, the consumptions of were considered in exclusion criteria. Participants were also recommended to avoid consumption of supplements such as alpha-tocopherol (> 400 IU/d), omega-3 (> 2000 mg/d), and any alpha-linolenic fatty acid contained foods (such as flaxseed, walnut, canola oil and so forth) during 7 days run-in period and 8 weeks intervention. Weekly checklists of *BV* consumption composed of questions about the daily number of *BV* glasses, timing of consumption and any BV-related undesired events were provided by participants for the assessment of compliance. Who had consumed the total amount of *BV* juice less than 80% was excluded from the study. In addition, the amount of leftover juice was recorded for every participant separately at every 2 weeks besides collecting the checklist data.

Face-to-face interviews at baseline assessments and every two-week visits in addition to the run-in period were carried out. *BV* juice had been provided by Takdaneh Agro-Industrial Company (Takdaneh, Co. Ltd., Marand, East-Azerbaijan, Iran). Each serving (500 ml) contained 55 g carbohydrates, 600 mg protein, 10 mg fat, 50 mg calcium, 100 mg sodium and 80 mg vitamin C. The berberine concentration in *BV* juice measured at 418 nm by means of the ultraviolet spectrophotometric method [30]. The average berberine level was 2.03 ± 0.03 μM obtained after testing five different samples of *BV* juice (different tetra packs).

Participants in the placebo group consumed two glasses each contained 240 ml *BV* juice placebo per day. Every glass was consumed with lunch and dinner meals separately, during the 8-week period. The placebo juice was similar to *BV* juice contents of total calorie, vitamin contents, color, taste and the size of packs. The beverages were asked to be stored in a refrigerator at 4°^c^. Sequence generation and allocation concealment were listed and marked by the study designer and implemented by clinic personnel who were unaware of the allocations at the time of enrolment. In the present follow-up interventional groups, study participants, clinic care providers, who had been responsible to hand in plastic bags and laboratory assessors, were blinded to know the assigned task. The follow-up took place between May 2013 and December 2014.

After the enrolment, in the first few days, dietitian instructed the participants to record their intakes by means of a 3 days collection of 24-h dietary records (two at working business days and one on the coming weekend). The portion size of the foods was measured using household utensils. A validated food frequency questionnaire with 136 food items was used to assess the consistency of dietary data obtained from 24-h dietary records at baseline [31]. The sum of certain nutrient intake level was assessed by means of nutritionist IV software (version 3.5.2; 1994, N-Squared Computing, San Bruno, CA).

### Laboratory assessments

#### Sample preparation

Overnight fasting blood sample (8 ml) was collected in sterile vacutainers containing anticoagulant (EDTA, Vacuum Blood Collection Tube - Gel & Clot Activator Tube, AMIS Medical Co., China) at baseline and endpoint of the intervention. Blood sampling was not undertaken at 3rd untill the 5th days of menstruation cycle to avoid sampling at the patient’s follicular phase to adjust possibly the laboratory measures with less hormonal variations because of the menstrual cycle. The blood samples immediately were transferred on ice and centrifuged at 3000×g (20°^C^) for 10 min to separate serum supernatant. Sera samples were transferred into 1.5 ml microtubes and stored at − 70°^C^ freezer until analysis.

#### Sample analysis

Eligible patients (*n* = 85) randomly assigned to the intervention or placebo group. Only 80 patients completed the trial. Serum levels of IGF-1, IGFBP-1, and IGFBP-3 were measured by the method of enzyme-linked immunosorbent assay (ELISA). The specific ELISA kits used for the assessments were as follow: insulin by Monobind (Cat No: 5825–300; California, USA; pre-specified primary outcomes), IGF-1 by Diasource ImmunoAssays S.A. (Cat. No: KAP 1581, Belgium), IGFBP-1 by Asbach Medical Products (AMP) kit (Cat. No: 110-E38700, Germany) and IGFBP-3 by IBL kit (Cat. No: MG59131, Germany). All the measurements performed according to the manufacturer’s protocol. The internal coefficient variations (CV) were about 92%. Biochemical tests were undergone at the Drug Applied Research Center at Tabriz University of Medical Sciences, where it is under the quality control of National Reference Laboratory. The evaluation of each biomarker was performed at the same time in one laboratory run and in random order to attenuate systematic errors. The name of the patient was replaced by a numeric code for each sera sample in order to meet the blindness criteria in the procedure of laboratory analysis.

#### Total mRNA extraction

The mRNA extraction was performed using the RNX-plus kit (Sinaclone Bioscience, Karaj, Iran)) following phenol-chloroform based mRNA extraction from a homogenized whole blood sample. Chloroform was added to the solution and centrifuged at 10000 RPM for15 min. This step was repeated. The supernatant was mixed by isopropanol following precipitation with a high-salt solution. Finally, the pellet was dried, diluted and supplied at − 70 °C freezer.

#### Quantitative real-time reverse transcriptase-polymerase chain reaction (qRT-PCR)

The concentration of the purified mRNA was specified using Nanodrop ND-1000 (Nanodrop Technologies, DE, USA). The reaction of cDNA synthesis from total RNA was carried out using (Prime Script™ RT reagent Kit; Perfect Real-Time, Cat No: RR037A, Tokyo Japan)) following the manufacturer’s protocol. A total volume of 10 μL of reaction mixture contains 5 μl master-mix SYBR Green (Takara, Tokyo, Japan), 0.5 μL of each primer, PCR-grade distilled water, and template cDNA (average1μg/mL). The nucleotide sequences of the PCR primer pair for the gene of interest are shown in Additional file 1: Table S1. Cycle threshold (Ct) was measured using qRT-PCR (RochLightCycler®96 systems, Penzberg, Germany) and carried out in duplicate experiments. Fold change of expression was also computed according to 2^-ΔΔCt^ formula [32].

#### Quantification of berberine in *Berberis vulgaris* juice

The developed high-performance liquid chromatography (HPLC) method by Chen and Cheng was used for the quantification of berberine in *BV* juice (Takdaneh, Iran) [33]. The standard of berberine chloride was purchased from Sigma (USA). A Cecil® (Cambridge, UK) chromatographic system equipped with an 1100 series pump, a 2-channel ERC-3315 degasser, a 1200 CE detector UV-Vis and an interface box and an analytical HPLC column, Eurospher C18 with pre-column (5 mm, 250 × 4.6 mm, KNAUER, Germany), was used for analysis. The mobile phase was prepared by mixing 60% acetonitrile (Merck, Germany) in 0.1% phosphoric acid (stock solution (85%) purchased from Scharlau, Spain) and adjusting the pH to 6 using concentrated ammonia (Merck, Germany) solution. The flow rate was set at 1 mL min^− 1^, and 20 μl of samples were injected by Autosampler. Berberine was detected by UV detector at 267 nm (maximum wavelength). The experiment was performed at room temperature.

### Statistical analysis

Data analysis was performed using the SPSS software package (version 13.0; SPSS Inc.). Kolmogorov-Smirnov test and liner histogram were used to assess the normality of data of variable. Paired samples t-test was used to compare each test within a group of intervention at the endpoint and baseline. Independent sample t-test was performed to compare a variable between *BV* and placebo groups at each certain timeline of treatment. When the distribution of data of a variable was not normal, Wilcoxon signed-rank test for within-group and Mann-Whitney U t-test for between comparisons was applied. The absolute treatment effect was calculated by assessing the mean change in the variable from baseline to the 8 weeks of follow-up between the two arms of intervention and was tested by a repeated-measures linear mixed model. The relative effect is a term used to define the proportional change in the treatment group relative to that in the placebo group, and likewise OR, it can interpret the proportional change in the treatment group rather than the placebo group. The *P-value* < 0.05 was assumed to be statistically significant.

## Results

### Patient characteristics

**Fig. 2:**
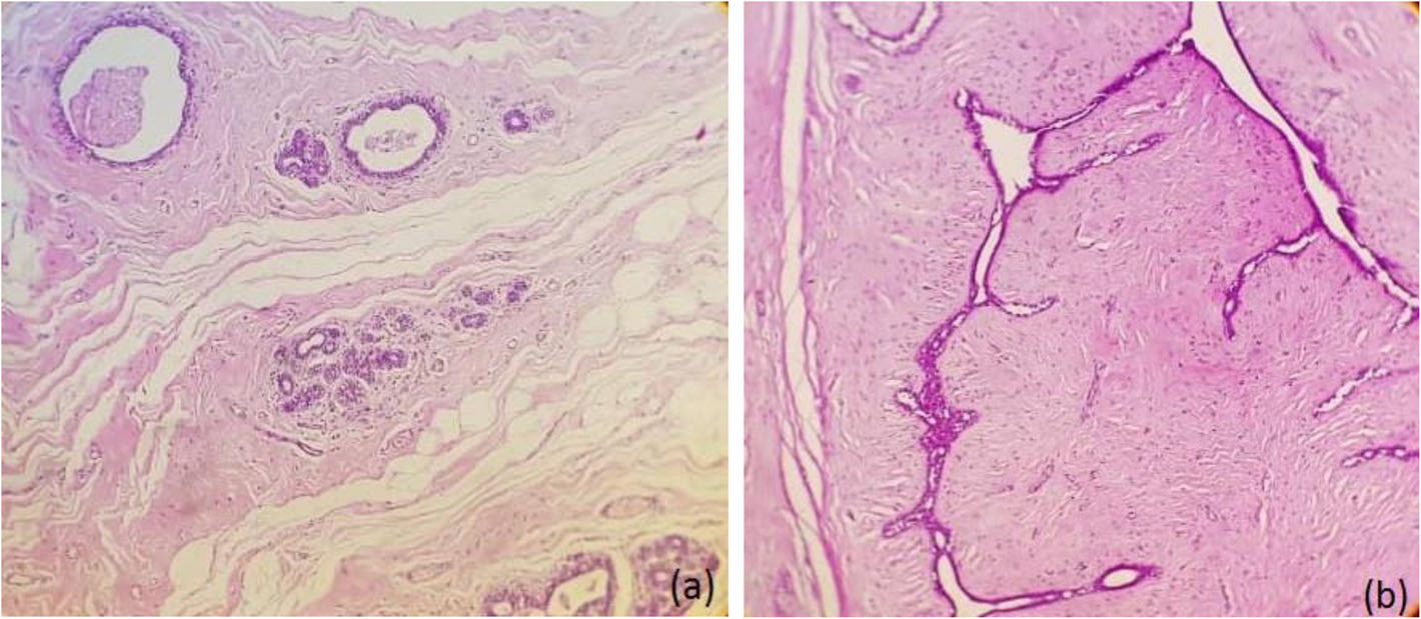
The typical microscopic images of histological sections of paraffin-embedded breast tissue diagnosed with fibrocystic (**a**) or fibroadenoma (**b**)

**Fig. 3:**
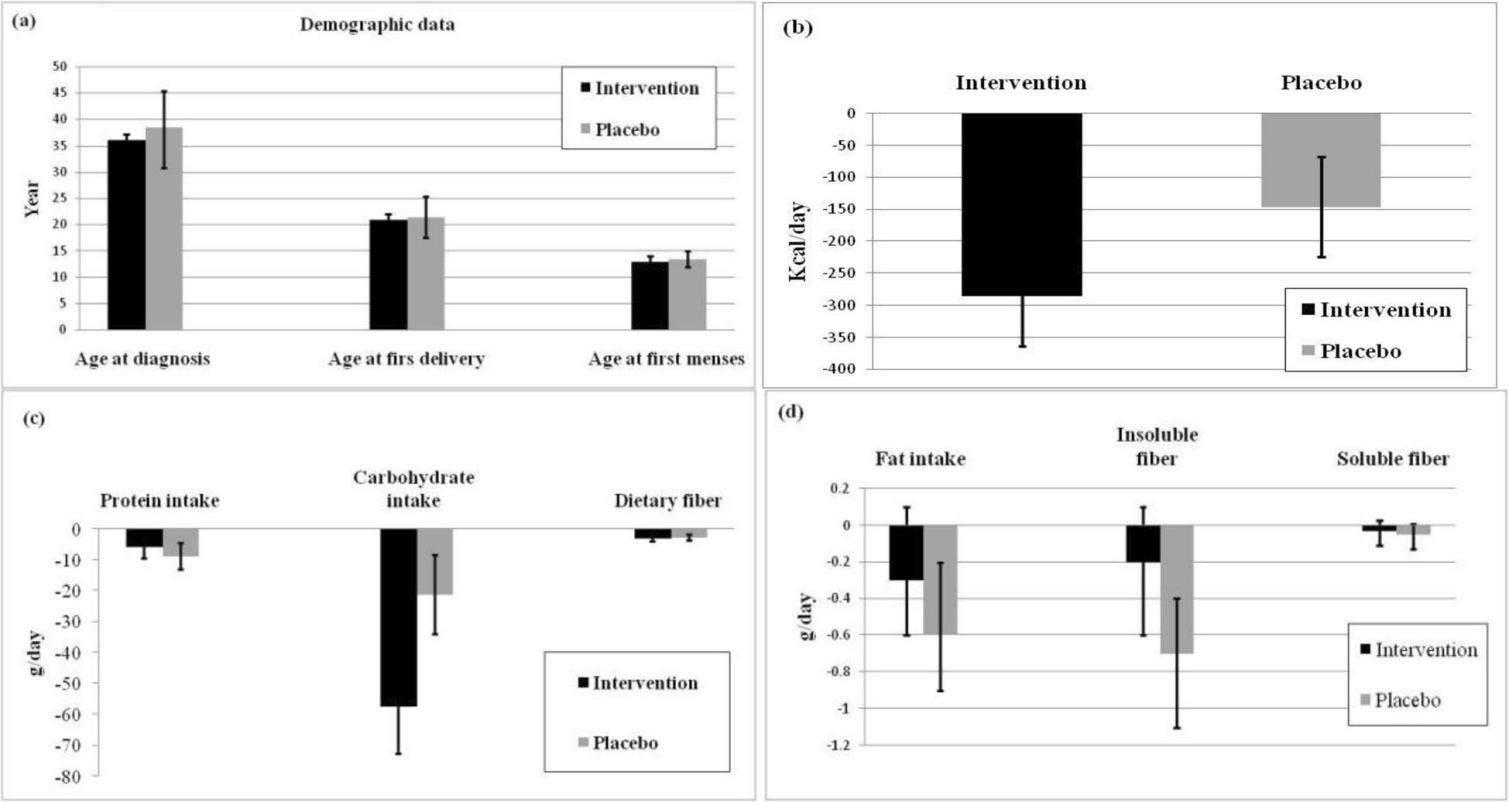
The averages of patients’ ages at baseline of intervention (**a**) and changes in daily calorie (**b**) and dietary intakes of nutrients (**c** and **d**) during post- and pre-tests of BBD patients either in *BV* juice placebo (placebo; *n* = 40) or *BV* juice (intervention; *n* = 40) groups. Independent sample t-test was performed

The excluded subjects during the enrollment and reasons for being non-respondent during the 8-week follow-up are depicted in the flow-chart diagram (Fig. 1). During the 8-week period of the intervention, 4 subjects in the intervention group and 1 in placebo failed to complete the trial. Of those in the intervention group, only one subject was lost to follow-up due to be non-respondent. At last, 40 participants have completed the treatments undergone at each interventional arm of this randomized trial. The average compliance rate was 94.3% in the intervention group and 90.7% in the placebo group. *BV* and its placebo juice did not cause any undesired and harmful side effects in patients during and over the 8 weeks trial. The minimum, maximum and median duration of follow-ups were 56, 65, and 59 days, respectively. The fibrocystic characteristic of the tumor (85%) was rather high in *BV* group than fibroadenoma subtype (15%). The typical microscopic images of histological sections of paraffin-embedded breast tissue diagnosed with fibrocystic or fibroadenoma were illustrated in Fig. 2. Fibrocystic subtype was also as common as 87.5% among participants in the placebo group while compared to fibroadenoma (12.5%) and they were different from frequencies observed in the BV group (*P* < 0.001). Pedigree analysis showed positive family history of BC in 12.5% of participants in the *BV* group, which was different from 25% of participants in the placebo group represented positive familial BC (*P* < 0.001) (Table 1). The demographic and general characteristics are summarized in Fig. 3. The mean changes in dietary intakes between the groups are presented in Fig. 3.

**Table 1:** Histopathologic characteristics, familial history and the status of multivitamin use among BBD patients in placebo (*n* = 40) and intervention (*n* = 40) groups at baseline of the study

Characteristics	Intervention (*n* = 40)	Placebo (*n* = 40)	*p-value*^a^
Histopathological characteristics			
Fibrocystic	34(85.0) ^b^	35(87.5)	< 0.001
Fibroadenoma	6(15.0)	5(12.5)	
Family history of BC			
No	35(87.5)	30(75.0)	< 0.001
Positive	5(12.5)	10(25.0)	
Family history of BBD			
No	40(100.0)	39(97.5)	< 0.001
Positive	0	1(2.5)	
Smoker			
Never	40(100)	40(100)	N/A
Ever	0	0	
Current	0	0	
Multivitamin use			
No	38(95.0)	35(87.5)	< 0.001
Yes	2(5.0)	5(12.5)	

*BC* breast cancer, *BBD* benign breast disorder

^a^Chi-square test was performed

^b^Data was expressed in the form of number of participants (relative frequency)

### The primary outcomes

The average plasma levels of IGF-1, IGFBP-1, and IGFBP-3 were compared within a group and between groups of either *BV* or placebo after 8 weeks of intervention in Table 2. The corresponding absolute and relative effects were also computed (Table 2). Plasma concentration of IGF-1 after 8 weeks treatment decreased significantly in both groups [Placebo: 187 ± 94 to 105 ± 5 ng/ml; (*P* < 0.001) and intervention: 193 ± 93 ng/ml to 87 ± 44 ng/ml; (*P* < 0.001)]. The relative treatment effect represented 16% drop in plasma IGF-1 concentration at *BV* group relative to placebo. The average plasma levels of IGFBP-1 increased in both *BV* and placebo groups during the interventions, but the observed changes failed to be statistically significant. There were remarkable decreases observed for plasma levels of IGFBP-3 both in placebo and *BV* groups (*P* < 0.001) (Table 2). The absolute treatment effect showed 111 ng/ml differences between mean changes of IGFBP-3 in the *BV* group and placebo, whereas this finding was not significant. The molar ratio of IGF-1 to IGFBPs (as IGF-1 bioavailability indices) in plasma of participants who underwent *BV* juice intervention and placebo juice were compared in Table 3 and the relative and absolute treatment effects were summarized as well. The molar ratio of IGF-1 to IGFBP-3 got raised after 8 weeks of treatment at both arms of the trial with no statically significant difference. Although the ratio of IGF-1 to 1GFBP-1 was apparently enhanced at both groups of intervention, the relative treatment effects indicated 37% fall at this ratio when comparing changes in *BV* group relative to changes observed in placebo. The plasma level of *PPAR-γ* increased at both groups though these changes were not significant.

**Table 2:** Serum level of growth factors and relative expression levels of the genes at baseline of study and 8 weeks after the intervention in women with BBT who received *BV* supplementation (*BV* group) versus placebo juice consumers

		Baseline (*n* = 80)				8-weeks follow-up (*n* = 80)			Absolute treatment effect			Relative treatment effects
Variable	n	Mean	S.D.	P^a^	n	Mean	S.D.	P^a^	Mean	95%CI	P^c^	
IGF-1^d^(ng/ml)												
Control	40	187.8	94.6	N/A	40	105.0	57.1	N/A	N/A	N/A	N/A	1.00
*BV*	40	193.1	93.2	0.80	40	87.6	44.3	0.13^b*^	−6.0	[(−32.4)-20.3]	0.411	0.84
IGFBP1^e^(ng/ml)												
Control	40	7.8	9.8	N/A	40	11.2	11.5	N/A	N/A	N/A	N/A	1.00
*BV*	40	5.9	5.8	0.30	40	9.5	13.3	0.55	−1.7	[(−5.12)-1.57]	0.295	1.03
IGFBP3^f^(ng/ml)												
Control	40	3454.3	937.8	N/A	38	1942.5	955.1	N/A	N/A	N/A	N/A	1.00
*BV*	40	3514.9	1127.7	0.79	40	2016.2	1215.7	0.76^*^	111.1	[(− 260)-480]	0.553	1.02
Relative gene expressions	n	Mean ^g^	S.E.M.	P^a^	n	Mean	S.E.M.	P^a^	Mean	95%CI	P^c^	
*PPAR-gamma*												
Control	26	8.03	3.62	N/A	34	5.08	1.42	N/A	N/A	N/A	N/A	N/A^**^
*BV*	33	0.72	0.37	0.05	33	4.27	3.58	0.83	−4.0	[(−10.74)-2.68]	0.233	N/A
*Hif-1α*												
Control	26	1.89	0.67	N/A	34	1.10	0.41	N/A	N/A	N/A	N/A	N/A
*BV*	27	1.41	0.47	0.56	23	0.54	0.19	0.23	−0.46	[(−1.80)-0.88]	0.495	N/A
*VEGF*												
Control	28	0.46	0.25	N/A	34	4.54	0.63	N/A	N/A	N/A	N/A	N/A
*BV*	37	5.85	2.87	0.07	23	1.90	0.62	0.68	3.52	[(−0.62)-7.66]	0.094	N/A

All data are expressed in geometric mean ± standard deviation (S.D.) and also mean ± standard error of the mean (S.E.M.). N/A: not applied to this model. The number of samples (n) was accompanied with some missings in evaluation the relative expression levels (2**ΔCt) because of extra Cts (> 40)

^*a*^ Independent sample t-test was performed between group

^*b*^ Paired t-test was performed to compare within changes in intervention group during the study. ^*^ Statistical significant difference in within group analysis. ^**^Variations caused not to calculate the relative treatment effects.^c^ Repeated measure of ANOVA was carried out in the main effect of model.^d^ Insulin-like growth factor binding protein (Log. transformed data was used in analysis).^e^ Insulin-like growth factor binding protein-1. ^f^ Insulin-like growth factor binding protein-3. ^g^ Data are expressed based on the values analyzed in independent-sample t-test.^f^ Relative expression levels was calculated based on 2 ^(−ΔCt)^. ^h^ Peroxisome proliferator-activated receptor-gamma. ^i^ Hypoxia-inducible factors. ^j^ Vascular edothelial growth factor

**Table 3:** The ratio of IGF-1 and IGFBPs at baseline of study and 8 weeks after the intervention in women with BBT who received *BV* supplementation (*BV* group) versus placebo juice consumers

	Baseline (*n* = 80)				8-weeks follow-up (*n* = 80)				Absolute treatment effect			Relative treatment effects
Variable	*n*	Mean	S.D.	P^a^	*n*	Mean	S.D.	P^a^	Mean	95% CI	P^c^	
IGF-1/IGFBP-3^d^												
Control	39	0.6	1.7	N/A	37	0.9	1.8	N/A	N/A	N/A	N/A	1.00
*BV*	39	0.3	0.4	0.86	40	0.6	1.0	0.18	−0.01	(−0.02–0.1)	0.72	0.99
IGF-1/1GFBP1^e^												
Control	39	90.6	101	N/A	39	38.1	66.1	N/A	N/A	N/A	N/A	1.00
*BV*	39	90.4	101	0.60	40	40.7	51.0	0.38^*^	0.90	(−25.8–27.6)	0.95	0.63
IGFBP-3/IGFBP-1^f^												
Control	39	1691.2	1709.6	N/A	37	757.9	1276.7	N/A	N/A	N/A	N/A	1.00
*BV*	39	1907.5	2429.4	0.70	40	1046.2	1593.1	0.37^*^	229.1	[(− 336.3)-794.4]	0.42	1.03
IGF-1/(IGFBP-3/IGFBP-1)												
Control	36	0.7	1.8	N/A	36	1.0	1.9	N/A	N/A	N/A	N/A	1.00
*BV*	39	0.4	0.4	0.39	39	0.6	1.1	0.27	−0.3	[(−0.8)-0.1]	0.14	1.16

All data are expressed in geometric mean ± S.D.. N/A: not applied to this model

^*a*^Independent sample t-test was performed between group

^*b*^Paired t-test was performed to compare within changes in intervention group during the study. ^*^ Statistical significant difference in within group analysis

^c^Repeated measure of ANOVA was carried out in the main effect of model

^d^Insulin-like growth factor to insulin-like growth factor binding protein 3 ratio

^e^Insulin-like growth factor to insulin-like growth factor binding protein 1 ratio

^f^Insulin-like growth factor binding protein 3 to insulin-like growth factor binding protein 1 ratio

**Fig. 4:**
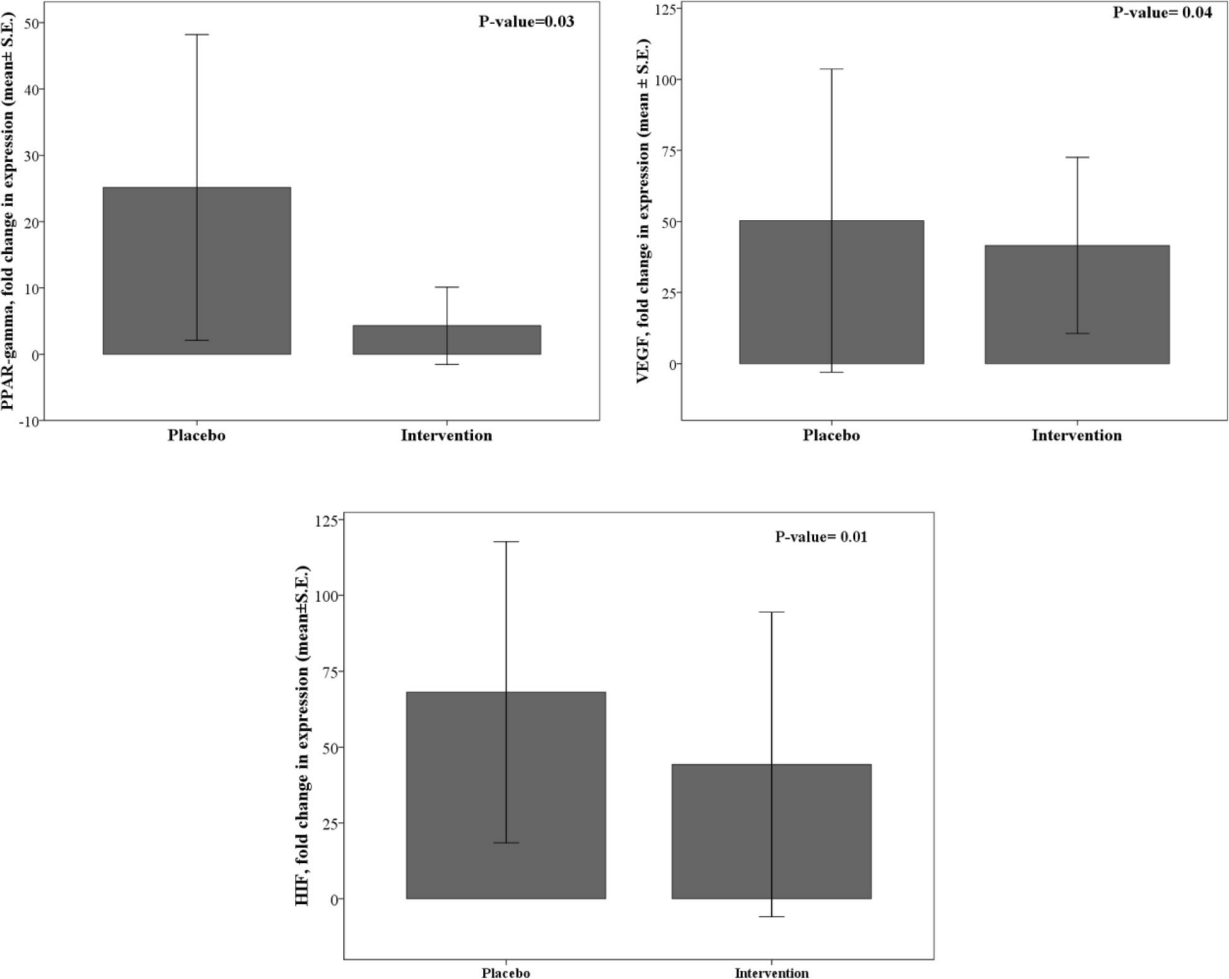
Fold changes in expression of *PPAR*-γ, *VEGF* and *HIF* genes between *BV* juice placebo and *BV* juice group

Figure 4 compares fold change in expression of *PPAR-γ*, *VEGF*, and *HIF-1α* between placebo and intervention groups. There were significantly higher levels of *PPAR-γ* and *VEGF* expressions in the placebo group than the intervention group. Furthermore, the intervention group attained fewer expression levels of *HIF* than the placebo group (*P* < 0.05) (Fig. 4).

**Fig. 5:**
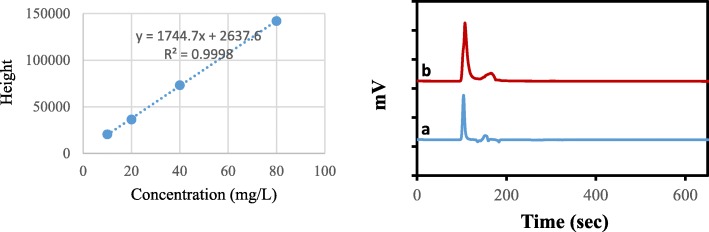
A linear correlation between the concentrations of berberine and measured peaks (r > 0.999). Chromatograms of berberine (40 μg mL-1) in the aqueous sample (**a**) and berberine in BV juice after 20 fold dilution by water (**b**)

A linear correlation between the concentrations of berberine and measured peaks was observed (r > 0.999; Fig. 5). Figure 5a shows the chromatograms of berberine (40 mg L^− 1^) in aqueous sample which prepared by dilution of stock solution. Figure 5b illustrates the chromatogram of berberine in *BV* juice after 20 fold dilution by water using the described HPLC method. Table 4 listed the concentrations of berberine in seven *BV* juice. Less varied concentrations of berberine were observed among 7 samples (1144 ± 126 mg L^− 1^). The relative standard deviation among studied samples was 11% showed an acceptable range of data using the criteria documented in FDA guideline to measure analytes [34].

**Table 4:** The concentration of berberine in seven *Berberis vulgaris* juices

Sample	Concentration (mg/L)
1	1099
2	1121
3	1420
4	1150
5	1101
6	1043
7	1075

## Discussion

**Fig. 6:**
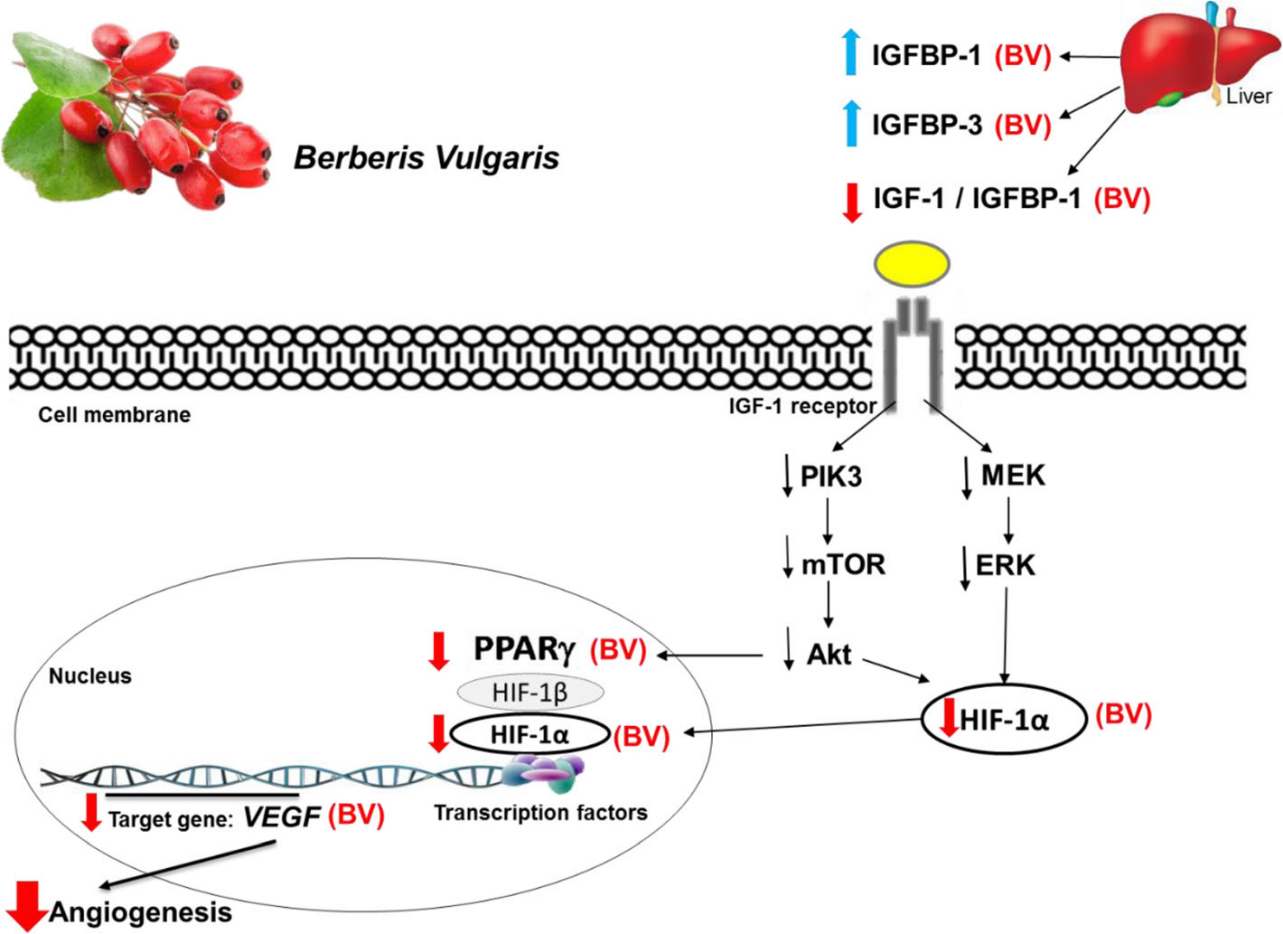
A summary diagram demonstrating the contribution of IGF-1/IGFBP to transcriptional levels of *PPAR-γ*, *VEGF*, and *HIF-1α* under the administration of *BV* intervention. Insulin-like growth factor-1 (IGF-1) is produced in hepatocytes, other tissues and tumor cells [3]. IGF-1 binds as a complex with IGF binding proteins (IGFBPs) in circulation to control IGF homeostasis and control IGF-1 signaling in target tissues where cells present transmembrane IGF-1 receptor [5]. Downstream IGF signaling was depicted in two pathways of phosphatidil-inositol-3-kinase/Akt (PI3K/Akt) and mitogen activating protein kinase/extracellular receptor kinase (MEK/ERK), can both induce angiogenesis [5, 36]. The activity of mediators in Ras/MAPK pathway can ultimately upregulate the transcription of genes involved in the proliferation of entails angiogenesis [5]. Consumption of BV juice contains berberine. Berberine led to enhanced plasma levels of IGFBP-1 and subsequently decreased IGF-1/IGFBP-1. Berberine can interact in different parts of PI3K/Akt pathway and MEK/ERK [34–36], suggesting as the main mechanism could explain down-regulating effects of BV juice on PPAR-γ, VEGF, and HIF-1α. Tested biomarkers were shown with BV colored by red

The core importance of IGF-1 in the pathogenesis of BBD which mediates the interaction between metabolic variables and the proliferation of breast benign neoplasm provides modifiable circumstances for life-style related interventions for which the area is widely remained to be resolved. This randomized clinical trial was implemented on BBD patients for the first time indicated that the intervening effects of *BV* juice can remarkably reduce plasma level of IGF-1 which is consistent with other experimental findings particularly pronounced for anti-diabetic effects of berberine [28, 35]. Kim et al. [36], indicated that the supplementations of berberine contained extract (*Scutellariabarbata D.Don* herb) inhibited leiomyoma cell growth through decreasing the expression levels of IGF-1 mRNA and its protein, suggesting a promising role for the regulation of IGF-1 dependent neoplastic changes and proliferation. In addition, it has been shown that treatment with berberine in 3 T3-L1 adipocytes enhanced glucose-stimulated insulin secretion via increasing insulin/IGF-1 signaling cascade [37]. The insulin/IGF-1 receptor mediates signaling through MAPK and phosphatidylinositol-4, 5-bisphosphate-3-kinase (PI3K)/Akt/mammalian target of rapamycin (mTOR) pathways to modulate cellular metabolism and growth (Fig. 6) [35]. Lamb and coworkers [38] showed that berberine supplementation (berberine sulfate trihydrate) in the content of a remedy (composed of berberine, vitamin D3, and vitamin K1) administered to postmenopausal women with metabolic syndrome, who were susceptible to develop osteoporosis, resulted in enhanced IGF-1 levels. Our findings suggested that daily regular *BV* juice consumption resulted in the improvement of circulating IGFBP-1 concentration, which has a principal role in controlling the free level of IGF-1 [5]. This finding is in line with Zhao et al. [35] showed that berberine as a constituent of extracts, caused an elevated level of *IGFBP-1* expression, possibly through increased insulin signaling. This could be partly explained by the regulatory effects of glucagon and glucocorticoids on the transcription of IGFBP-1, which is also suppressed by insulin [39]. Indeed, the alteration in IGFBP-1 is considered to be highly dependent on environmental factors such as dietary factors [40], whereas *IGFBP-3* is a growth hormone-dependent variable [3]. The increased expression and circulation level of IGFBP-1 induced by *BV* juice consumption might be explained through the mechanism underlying berberine as a ligand for PPAR-γ protein, which is a transcriptional factor for the expression of *IGFBP-1* gene [41]. Our findings showed that in spite of remarkable increases in plasma IGFBP-1 levels in response to regular daily usage of *BV* juice, plasma IGFBP-3 diminished after 8 weeks of intervention. Several findings have suggested that the high circulating levels of IGFBP-3 could attenuate the growth promoting effects of IGF-1 [3, 42]. On the other hand, it is pronounced that the increasing levels of IGFBP-3 seems as a proliferative factor and likely associated with poorer prognosis in malignancy [3, 6, 42]. This might be explicated by the pool of bound IGF-1 to IGFBP-3 which could act as a considerable reservoir to release IGF-1 in a turn-over process depends regularly on tumoral uncontrolled growth [6]. Accordingly, Su et al. [3] indicated that the elevated levels of IGFBP-3 might associate with proliferative benign breast disease. Given that the up-regulation of *IGFBP-1* gene is sensitively influenced by *BV* intervention rather than less promptly affected IGFBP-3 in responses to *BV* juice intake. Although our findings on *BV* supplementation suggested significant decreases in the plasma IGF-1/IGFBP-1 M ratio to constrain free IGF-1 bioavailability index [43], the *BV* treatment effects on IGF-1/IGFBP-3 remained unchanged relative to placebo. On the other hand, prior studies have denoted the importance of circulation IGF1/IGFBP-3 M ratio in almost different malignancies [44], such as breast cancer [42]. Enriori et al. [45] warranted to estimate the predictive value of IGFBP-3 in benign breast disease. Although they assumed that both IGFBP-1 and IGFBP-3 may form a network to reduce the growth promoting effect of free IGF-1, they did not introduce a priority or range of the functionality for different types of IGFBPs. Perhaps, the affinity of IGF-1 to different IGFBPs are different and could be reasoned out in this regard. However, evidence connecting the elevated levels of IGFBP-3 to the risk of later onset of breast cancer development is conflicting [3, 8]. In our study, the IGF-1/IGFBP-3 ratio increased at both intervention and placebo groups, which might indicate the weakness of IGFBP-3 to compensate the binding capacity to free form of IGF-1. However, it is additionally addressed that IGF-1/IGFBP-3 ratio is not a suitable marker for measuring free IGF-1 [6]. In conclusion, there might be a possible biological mechanism to explain the apparently decreased levels of IGFBP-3 by enhancing peripheral insulin levels or its sensitivity [8]. Aljada et al. [46] indicated that metformin can reduce hyperinsulinemia, subsequently may lead to an elevated level of IGFBP-1. They also showed that metformin therapy resulted in the reduction of IGF-1/IGFBP1 ratio. Thereby, based on the former evidence put forth concepts suggesting that IGFBP-1 likewise IGFBP-3 is capable to bind IGF-1 with potent affinity [6], has given rise to the notion that *BV* juice supplementation led to better effectiveness on IGFBP-1 variations rather than IGFBP-3 in terms of IGF-1 bioavailability in the circulation of BBD patients. Though several studies collectively have appealed for the important role of IGFBP on cancer treatment based on targeting the IGFBP-regulated cancer pathways as cancer biomarkers, some have denoted that the varied expression of IGFBP subtypes in different tumor tissues could better provide information on disease prognosis and treatment responsiveness in future follow-up studies [6, 8].

It has been shown that berberine could improve the FFA-induced insulin resistance by reducing the expression of *PPAR-γ* [47], which is in agreement with our study showed that *BV* juice consumption can significantly reduce the fold change in gene expression of *PPAR-γ* (Fig. 6). Although Zhou et al. indicated that the expression of *PPAR-γ* upregulated in diabetic adipocytes [48], they suggested that berberine may decrease *PPAR-γ* mRNA and protein levels in 3 T3-L1 cells. In addition, Pham et al. [49] showed that berberine may induce the repression of adipogenic markers such as C/EBPa and *PPAR-γ*. Recent studies have accordingly suggested that the low expression levels of *PPAR-γ* may also associate with the inhibition of fatty acid uptake, adipogenesis and alleviating insulin resistance [48, 49]. Feng et al. [50] illustrated that berberine can inhibit COX-2 expression in rat small intestinal mucosa through influencing the expression of *PPAR-γ*, meantime berberine was found to decrease LPS-induced PPAR-γ production by reducing *PPAR-γ* expression.

Therefore, it is noticed that berberine varies *PPAR-γ* expression dependent on cell and tissue types [51]. It is illustrated that *PPAR-γ* overexpressed in ERBB2-positive breast cancer cells [52]. Moreover, Keller et al. [53] indicated that PPAR-γ may have an anti-estrogenic role through inducing inhibition on the binding affinity of ER protein with downstream target genes. Gao et al. [54] indicated that berberine reduced palmitate-induced lipoapoptosis and caused an increment of glucose-stimulated insulin levels in HIT-T15 cells, through increased *PPAR*-γ expression. While another experimental study showed that berberine intervention in mice with high-fat-diet-induced obesity can reduce adiposity, food intake and serum levels of glucose, triglyceride and total cholesterol may associate with the down-regulation of *PPAR-γ* expression [55]. However, little is known about clinicopathological traits related to the status of PPAR-γ in the benign diseases of the breast. Therefore, the biological importance of PPAR-γ remains largely undetermined in benign breast disease.

It had been demonstrated that berberine can decrease angiogenesis and related biomarkers including *VEGF* in breast cancer cells [56]. It has been suggested that berberine treatment in lung cancer cells inhibited *HIF* and *VEGF* expressions [27]. These findings further support the idea of our study that *HIF* expression decreased after 8 weeks of intervention (Fig. 6).

Some limitations exist in the present study. The length of study (8 weeks) was almost short, while growth factor levels may need longer period of time in interventional studies. Albeit the inter-individual genetic variations related to IGF-1 and IGFBPs metabolism such as possible polymorphism could be a source of variation for a biomarker, randomization in the group assignment may help to attenuate the probability of comparable polymorphic co-effects. The protein levels of PPAR-γ, VEGF and HIF were not measured and considered as a limitation. The absorption of berberine is very low [57, 58]. Therefore, the plasma levels do not seem remarkable measures to indicate berberine-related pharmacologic effects [57]. However, the tissue concentrations of berberine were documented to be higher than plasma levels [57, 58] and seem to be varied less by plasma levels. Though, the biopsy samples of BBD were not available to test the tissue concentration of berberine. Women on hormonal replacement therapy and who received medicine to control hyperinsulinemia under insulin-resistant conditions were excluded, but other intervening pharmacologic factors could make variations in primary measured variables.

## Conclusions

This interventional study showed the effectiveness of *BV* juice supplementation on changes in IGF and IGF-bioavailability in women with BBD. Our findings showed that *BV* administration resulted in enhanced IGFBP-1 level and reduced IGF-1/IGFBP-1 ratio which both suggest that *BV* juice might potentially reduce IGF-dependent tumor progression toward malignancy from benign breast disease. In addition, *BV* juice intervention led to reduction in percent changes of *PPAR-γ*, *VEGF* and *HIF* expressions, highlighting the possible inhibitory effects of *BV* juice on malignant transformation for BBD. Our findings provide new insights into the priority of IGFBP-1 rather than IGFBP-3 to become influenced by *BV* juice supplementation. In overall, *BV* juice supplementation demonstrated promising properties in terms of IGF-1 related bioavailability indices and transcription of candidate genes related to tumorigenesis. IGFBP responsiveness and downstream transcriptional variations in favor of controlling the malignancy transformation by dietary intervention is warranted for future studies in patients with benign neoplastic tissues.

## Supplementary information

**Additional file 1: Table S1.** Nucleotide sequences of primers used in real-time PCR.

## Data Availability

The data that support the findings of this study are available from Tabriz University of Medical Sciences but restrictions apply to the availability of these data, which were used under license for the current study, and so are not publicly available. However, data are available from the authors upon reasonable request and with permission of Tabriz University of Medical Sciences.

## Abbreviations

ABCG2: ATP-binding cassette sub-family G member 2
BBD: Benign breast disease
BC: Breast cancer
*BV*: *Berberis vulgaris*
HIF-1α: Hypoxia-inducible factor
HRT: Hormone replacement therapy
IGF: Insulin-like growth factor
IGFBP: Insulin-like growth factor binding protein
IRCT: Iranian Registry of Clinical Trials
OR: Odds ratio
PPAR: Peroxisome-proliferative activating receptor
RT: Relative treatment
VEGF: Vascular endothelial growth factor
WHO: World Health Organization

## References

[1] Hartmann LC, Sellers TA, Lingle WL, Degnim AC, Vierkant RA, Maloney SD (2005). Benign breast disease and the risk of breast cancer. N Engl J Med.

[2] Griffin JL, Pearlman MD (2011). Primer on the management of benign breast diseases. Breast Health.

[3] Su X, Colditz GA, Willett WC, Collins LC, Schnitt SJ, Connolly JL (2010). Genetic variation and circulating levels of IGF-I and IGFBP-3 in relation to risk of proliferative benign breast disease. Int J Cancer.

[4] Lagiou P, Samoli E, Lagiou A, Zourna P, Barbouni A, Georgila C (2013). A comparison of hormonal profiles between breast cancer and benign breast disease: a case–control study. Ann Oncol.

[5] Zhang X, Yee D (2002). Insulin-like growth factor binding protein-1 (IGFBP-1) inhibits breast cancer cell motility. Cancer Res.

[6] Baxter RC (2014). IGF binding proteins in cancer: mechanistic and clinical insights. Nat Rev Cancer.

[7] Lofqvist C, Chen J, Connor KM, Smith ACH, Aderman CM, Liu N (2007). IGFBP3 suppresses retinopathy through suppression of oxygen-induced vessel loss and promotion of vascular regrowth. Proc Natl Acad Sci U S A.

[8] Pollak M (2012). The insulin and insulin-like growth factor receptor family in neoplasia. Nat Rev Cancer.

[9] Webb SJ, Geoghegan TE, Prough RA, Michael Miller KK (2006). The biological actions of dehydroepiandrosterone involves multiple receptors. Drug Metab Rev.

[10] Renehan AG, Frystyk J, Flyvbjerg A (2006). Obesity and cancer risk: the role of the insulin-IGF axis. Trends Endocrinol Metab.

[11] Lehrke M, Mitchell AL (2005). The many faces of PPARγ. Cell..

[12] Saez E, Rosenfeld J, Livolsi A, Olson P, Lombardo E, Nelson M (2004). PPAR gamma signaling exacerbates mammary gland tumor development. Genes Dev.

[13] Salven P, Perhoniemi V, Tykk H, Joensuu H (1999). Serum VEGF levels in women with a benign breast tumor or breast cancer. Breast Cancer Res Treat.

[14] Pal A, Vernon BL, Nikkhah M (2018). Therapeutic neovascularization promoted by injectable hydrogels. Bioactive Materials.

[15] Adams J, Carder PJ, Downey S, Forbes MA, MacLennan K, Allgar V (2000). Vascular endothelial growth factor (VEGF) in breast cancer: comparison of plasma, serum, and tissue VEGF and microvessel density and effects of Tamoxifen. Cancer Res.

[16] Fukumura D, Kloepper J, Amoozgar Z, Duda DG, Jain RK (2018). Enhancing cancer immunotherapy using antiangiogenics: opportunities and challenge. Nat Rev Clin Oncol.

[17] Xin X, Rodrigues M, Umapathi M, Kashiwabuchi F, Ma T, Babapoor-Farrokhran S (2013). Hypoxic retinal Müller cells promote vascular permeability by HIF-1–dependent up-regulation of angiopoietin-like 4. PNAS..

[18] Gilkes DM (2013). Role of hypoxia-inducible factors in breast cancer metastasis. Future Oncol.

[19] Dales J, Beaufils N, Silvy M, Picard C, Pauly V, Pradel V (2010). Hypoxia inducible factor 1a gene (HIF-1a) splice variants: potential prognostic biomarkers in breast cancer. BMC Med.

[20] Fatehi M, Saleh TM, Fatehi-Hassanabad Z, Farrokhfal KH, Jafarzadeh M, Davodi S (2005). A pharmacological study on Berberis vulgaris fruit extract. J Ethnopharmacol.

[21] Javadzadeh M, Ebrahimi A (2013). The traditional uses and pharmacological effects of different parts Berberis Vulgaris (berberine) in Iran. Sci Agric.

[22] Kim JB, Yu JH, Ko E, Lee KW, Song AK, Park SY (2010). The alkaloid Berberine inhibits the growth of Anoikis-resistant MCF-7 and MDA-MB-231 breast cancer cell lines by inducing cell cycle arrest. Phytomedicine..

[23] Eom KS, Kim HJ, So HS, Park R, Kim TY (2010). Berberine-induced apoptosis in human glioblastoma T98G cells is mediated by endoplasmic reticulum stress accompanying reactive oxygen species and mitochondrial dysfunction. Biol Pharm Bull.

[24] Imanshahidi M, Hosseinzadeh H (2008). Pharmacological and therapeutic effects of Berberis vulgaris and its active constituent, berberine. Phytother Res.

[25] Kim JB, Ko E, Han W, Shin I, Park SY, Noh DY (2008). Berberine diminishes the side population and ABCG2 transporter expression in MCF7 breast cancer cells. Planta Med.

[26] Hamsa TP, Kuttan G (2012). Antiangiogenic activity of berberine is mediated through the downregulation of hypoxia-inducible factor-1, VEGF, and proinflammatory mediators. Drug Chem Toxicol.

[27] Lingyi F, Wangbing C, Wei G, Jingshu W, Yun T, Dingbo S (2013). Berberine targets AP-2/hTERT, NF-κB/COX-2, HIF-1α/VEGF and cytochrome-c/caspase signaling to suppress human cancer cell growth. Plose one.

[28] Gu Y, Zhang Y, Shi X, Li X, Hong J, Chen J (2010). Effect of traditional Chinese medicine berberine on type 2 diabetes based on comprehensive metabonomics. Talanta..

[29] Association WM (2013). Declaration of Helsinki: ethical principles for medical research involving human subjects. JAMA..

[30] Shamsa F, Monsef H, Ghamooshi R, Verdian-rizi M (2008). Spectrophotometric determination of total alkaloids in some Iranian medicinal plants. Thai J Pharm Sci.

[31] Pirouzpanah S, Taleban FA, Mehdipour P, Atri M, Hooshyareh-rad A (2014). The biomarker-based validity of a food frequency questionnaire to assess the intake status of folate, pyridoxine and cobalamin among Iranian primary breast cancer patients. Eur J Clin Nutr.

[32] Pfaffl MW (2001). A new mathematical model for relative quantification in real-time RT–PCR. Nucleic Acids Res.

[33] Chen CM, Chang HC (1995). Determination of berberine in plasma, urine and bile by high-performance liquid chromatography. J Chromatogr B Biomed Appl.

[34] Bansal S, DeStefano A (2007). Key elements of bioanalytical method validation for small molecules. AAPS J.

[35] Zhao HL, Sui Y, Qiao CF, Yip KY, Leung RKK, Tsui SKW (2012). Sustained antidiabetic effects of a berberine-containing chinese herbal medicine through regulation of hepatic gene expression. Diabetes..

[36] Kim DI, Lee TK, Lim IS, Kim H, Lee YC, Kim CH (2005). Regulation of IGF-I production and proliferation of human leiomyomal smooth muscle cells by Scutellaria barbata D. Don in vitro: isolation of flavonoids of apigenin and luteolon as acting compounds. Toxicol Appl Pharmacol.

[37] Ko BS, Choi SB, Park SK, Jang JS, Kim YE, Park S (2005). Insulin sensitizing and insulinotropic action of berberine from Cortidis rhizoma. Biol Pharm Bull.

[38] Lamb JJ, Holick MF, Lerman RH, Konda VR, Minich DM, Desai A (2011). Nutritional supplementation of hop rho iso-alpha acids, berberine, vitamin D3, and vitamin K1 produces a favorable bone biomarker profile supporting healthy bone metabolism in postmenopausal women with metabolic syndrome. Nutr Res.

[39] Suwanichkul A, Allander SV, Morris SL, Powell DR (1994). Glucocorticoids and insulin regulate expression of the human gene for insulin-like growth factor-binding protein-1 through proximal promoter elements. J Biol Chem.

[40] Crowe FL, Key TJ, Allen NE, Appleby PN, Overvad K, Grønbæk H (2009). The association between diet and serum concentrations of IGF-I, IGFBP-1, IGFBP-2, and IGFBP-3 in the European prospective investigation into Cancer and nutrition. Cancer Epidemiol Biomark Prev.

[41] Degenhardt T, Matilainen M, Herzig KH, Dunlop TW, Carlberg C (2006). The insulin-like growth factor-binding protein 1 gene is a primary target of peroxisome proliferator-activated receptors. J Biol Chem.

[42] Rosendahl AH, Hietala M, Henningson M, Olsson H, Jernström H (2011). IGFBP1 and IGFBP3 polymorphisms predict circulating IGFBP-3 levels among women from high-risk breast cancer families. Breast Cancer Res Treat.

[43] Zumkeller W (2001). IGFs and IGFBPs: surrogate markers for diagnosis and surveillance of tumour growth?. J Clin Pathol.

[44] Gao Y, Katki H, Graubard B, Pollak M, Martin M, Tao Y (2012). Serum IGF1, IGF2 and IGFBP3 and risk of advanced colorectal adenoma. Int J Cancer.

[45] Enriori PJ, Fischer CR, Gor JR, Etkin AE, Calandra RS, Luthy IA (2003). Augmented serum levels of the IGF-I/IGF-binding protein-3 ratio in pre-menopausal patients with type I breast cysts. Eur J Endocrinol.

[46] Aljada AMS (2012). Metformin and neoplasia: implications and indications. Pharmacol Ther.

[47] Chen Y, Li Y, Wang Y, Wen Y, Sun C (2009). Berberine improves free-fatty-acid–induced insulin resistance in L6 myotubes through inhibiting peroxisome proliferator–activated receptor γ and fatty acid transferase expressions. Metab..

[48] Zhou J, Zhou SH (2010). Berberine regulates peroxisome proliferator-activated receptors and positive transcription elongation factor b expression in diabetic adipocytes. Eur J Pharmacol.

[49] Pham TPT, Kwon J, Shin J (2011). Berberine exerts anti-adipogenic activity through up-regulation of C/EBP inhibitors, CHOP and DEC2. Biochem Biophys Res Commun.

[50] Feng AW, Gao W, Zhou GR, Yu R, Li N, Huang XL (2012). Berberine ameliorates COX-2 expression in rat small intestinal mucosa partially through PPARγ pathway during acute endotoxemia. Int Immunopharmacol.

[51] Yin J, Zhou SW, Zhang KB, Tang JL, Guang LX, Ying Y (2008). Chronic effects of berberine on blood, liver glucolipid metabolism and liver PPARs expression in diabetic hyperlipidemic rats. Biol Pharm Bull.

[52] Kourtidis A, Srinivasaiah R, Kourtidis A, Srinivasaiah R, Carkner RD, Brosnan MJ (2009). Peroxisome proliferator-activated receptor-γ protectsERBB2-positive breast cancer cells from palmitate toxicity. Breast Cancer Res Treat.

[53] Keller H, Givel F, Perroud M, Wahli W (1995). Signaling cross-talk between peroxisome proliferator-activated receptor/retinoid X receptor and estrogen receptor through estrogen response elements. Mol Endocrinol.

[54] Gao N, Zhao TY, Li XJ (2011). The protective effect of berberine on β-cell lipoapoptosis. J Endocrinol Investig.

[55] Hu Y, Davies GE (2010). Berberine inhibits adipogenesis in high-fat diet-induced obesity mice. Fitoterapia..

[56] Kim S, Oh S, Lee J, Han J, Jeon M, Jung T (2013). Berberine suppresses TPA-induced fibronectin expression through the inhibition of VEGF secretion in breast cancer cells. Cell Physiol Biochem.

[57] Imenshahidi M, Hosseinzadeh H (2016). Berberis Vulgaris and berberine: an update review. Phytother Res.

[58] Pirillo A, Catapano AL (2015). Berberine, a plant alkaloid with lipid- and glucose-lowering properties: from in vitro evidence to clinical studies. Atherosclerosis..

