# Supplementary material for: The effects of Berberis vulgaris consumption on plasma levels of IGF-1, IGFBPs, PPAR-γ and the expression of angiogenic genes in women with benign breast disease: a randomized controlled clinical trial
Source: BMC Complement Altern Med. 2019 Nov 21;19:324. doi: 10.1186/s12906-019-2715-1 (PMC6868871; doi:10.1186/s12906-019-2715-1)
Supplement: Supplementary file 1 — Additional file 1: Table S1. Nucleotide sequences of primers used in real-time PCR. [file 12906_2019_2715_MOESM1_ESM.docx]

| **Table S1.** Nucleotide sequences of primers used in real-time PCR. | | | | |
| --- | --- | --- | --- | --- |
| **Gene** | **Forward primer** | **Reverse primer** | **Product size (bp)** | **Accession number** |
| *HIF-1α* | 5’-TCTGCAACATGGAAGGTATTGC-3’ | 5’-ACCAAGCAGGTCATAGGTGGTT-3’ | 107 | NM_001243084.1 |
| *PPAR-γ* | 5’-AGTGGGGATGTCTCATAATGCC-3’ | 5’ -AGGTCAGCGGAC TCTGGATTC-3’ | 110 | NM_015869.4 |
| *VEGF* | 5’-CTACCTCCACCATGCCAAGT-3’ | 5’-CCACTTCGTGATGATTCTGC-3’ | 74 | NM_001025366.2 |
| *HGPRT* | 5’-TGGACAGGACTGAACGTCTTG-3’ | 5’- CCAGCAGGTCAGCAAAGAATTTA-3’ | 111 | NM_000194([3](#_ENREF_3)) |
| *HIF-1α*, *hypoxia inducible factor-1α*; *PPAR*-*γ*, *peroxisome-proliferative activating receptor; VEGF*, *vascular endothelial growth factor*; *HGPRT*, *hypoxanthine-guanine phosphoribosyltransferase*; bp, base pair*.* | | | | |
